# Effects of Dietary Interventions on Nutritional Status in Patients with Gastrointestinal Cancers: A Systematic Review

**DOI:** 10.3390/biomedicines14010240

**Published:** 2026-01-21

**Authors:** Camelia Maria Caragescu (Lup), Laura Grațiela Vicaș, Angela Mirela Antonescu, Nicole Alina Marian, Octavia Gligor, Mariana Eugenia Mureșan, Patricia-Andrada Grigore, Eleonora Marian

**Affiliations:** 1 Doctoral School of Biomedical Sciences, University of Oradea, No. 1 University Street, 410087 Oradea, Romania; caragescucamelia@ymail.com (C.M.C.); mariannicole@ymail.com (N.A.M.); mmuresan@uoradea.ro (M.E.M.); emarian@uoradea.ro (E.M.); 2 Department of Pharmacy, Faculty of Medicine and Pharmacy, University of Oradea, No. 29 Nicolae Jiga Street, 410028 Oradea, Romania; 3 Department of Preclinical Disciplines, Faculty of Medicine and Pharmacy, University of Oradea, 10 Piata 1 Decembrie Street, 410073 Oradea, Romania; aantonescu@uoradea.ro (A.M.A.); octavia.gligor@uoradea.ro (O.G.); 4 “Saint John” Emergency Clinical Hospital, Vitan-Bârzești Street 13, 042122 Bucharest, Romania; resteapatri@yahoo.com

**Keywords:** malnutrition, digestive cancers, gastric cancer, colorectal cancer, clinical nutrition, nutritional support, oral supplements, enteral nutrition

## Abstract

**Introduction/Object**: Gastrointestinal cancers are among the most common types of neoplasms and are often associated with malnutrition, which affects physical performance, treatment tolerance and prognosis. This paper aims to synthesize, through a systematic search, the evidence on the impact of nutritional interventions on nutritional status in patients with digestive cancers prone to malnutrition. **Methods**: A systematic search was performed in PubMed, MDPI, Web of Science and ScienceDirect, for articles published between 2009 and 2025. Overall, 14,503 records were identified, and after screening of titles, abstracts and full-text evaluation, 80 studies (cross-sectional and cohort) were included. Data extraction was performed by a single researcher, using pre-established criteria and a standardized table, and the assessment of study quality was performed qualitatively, taking into account study design, sample size, nutritional assessment methods and clarity of reporting of results. **Results**: Evidence suggests that individualized and early applied nutritional interventions contribute to maintaining weight and protein status, improve tolerance to oncological treatments and may positively influence patient survival. **Conclusions**: Nutritional therapy plays a crucial role in preventing complications and supporting the body during oncological treatment, optimizing patients’ quality of life. This review provides a clear synthesis of the current evidence and recognizes methodological limitations related to the qualitative assessment of the included studies.

## 1. Introduction

**Figure 1:**
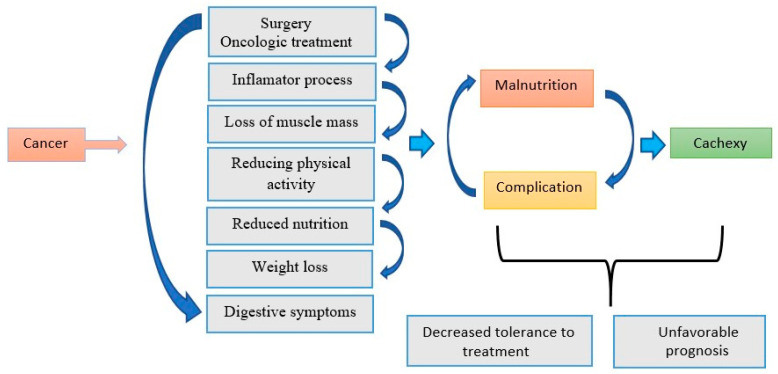
Mechanism of malnutrition in digestive cancers.

Digestive tract cancers are the most common types of cancer, and their incidence is constantly increasing [1]. Patients diagnosed with cancer are at increased risk of malnutrition, firstly due to the location of the tumor and secondly due to the complexity of oncological treatment that includes chemotherapy, radiotherapy and extensive surgery [2]. Once malnutrition is established, the negative consequences on the entire body are quite serious, negatively influencing the treatment. The result is loss of muscle mass, decreased physical performance, and diminished emotional state and quality of life [3]. The diagnosis of malnutrition is based on involuntary weight loss, a low body mass index (BMI), low muscle mass, insufficient food intake and the presence of the inflammatory process [4]. Nutritional counseling is important in preventing and correcting malnutrition. It is based on personalized nutritional interventions, through oral nutritional supplements, enteral or parenteral nutrition [5]. Adequate nutrition can reduce possible postoperative complications, such as wound infections and mortality [6]. During chemotherapy, specialized enteral nutrition helps with protein absorption and limits adverse effects, such as oral mucositis [7]. Following gastrectomy, weight loss can jeopardize compliance with adjuvant oncological treatment and is associated with poor survival [8]. If oral intake becomes insufficient, nutrition administration via nasogastric tube is required [9]. Regardless of the level of nutrition, malnutrition can set in and is associated with treatment interruption, unplanned admissions and prolonged hospital stay [10]. When oral nutrition does not cover at least 50% of the requirement for a prolonged period or is not possible due to treatment, nutritional status or tumor location, artificial nutrition is considered, and the choice between enteral and parenteral nutrition is based on the clinical situation, therapeutic plan and prognosis of the patient [11]. Sarcopenia and systemic inflammation affect the modification of protein and carbohydrate metabolism, favoring the loss of muscle mass [12], while adequate nutrition after surgery helps to speed up discharge and reduces the risk of complications [13]. In the case of elderly patients, integrated assessment of nutritional status is essential to prevent malnutrition [14], especially since they may develop cancer cachexia, characterized by progressive loss of muscle mass and associated with increased morbidity and mortality, which cannot be completely corrected by conventional nutritional support [15]. Thus, diet represents an important modifiable factor in the prevention and management of chronic diseases [16], and lifestyle and nutrition positively influence the quality of life and the level of post-treatment fatigue, even in the context of increasing survival of patients with colorectal cancer [17]. Frequently in hospitalized patients, poor nutritional status influences the results of surgical interventions and the effectiveness of therapy [18], but the adoption of healthy dietary strategies reduces inflammatory markers and the risk of complications [19], and nutritional support, even when suboptimal, brings significant benefits to undernourished patients [20]. Malnutrition associated with digestive tract cancers has multiple causes, including the underlying disease, oncological therapy and metabolic changes, which influence both treatment tolerance and patient prognosis [21]. Understanding the mechanisms of malnutrition is essential for the development of nutritional interventions and therapeutic strategies in patients with digestive tract cancer. Our study aims to analyze the impact of dietary interventions on nutritional status, emphasizing the importance of a personalized approach to optimize clinical outcomes [22]. To highlight the complex interaction between cancer, treatments, and malnutrition, Figure 1 presents the mechanisms by which these factors contribute to the development of cachexia and nutritional complications.

## 2. Methods

### 2.1. Study Design

The systematic review was carried out following the Preferred Reporting Items for Systematic Reviews and Meta-Analysis (PRISMA) guidelines (Table S1), PROSPERO ID 1268270.

### 2.2. Search Strategy

The search strategy was developed based on the fundamental concepts of the research question, including gastrointestinal cancers, nutritional status, malnutrition, sarcopenia, cachexia and nutritional interventions. Relevant keywords and, where applicable, controlled vocabulary were used, and Boolean operators were used to optimize the sensitivity of the search.

The systematic search was performed in the databases PubMed, ScienceDirect, MDPI and ResearchGate, selected for their extensive coverage of the biomedical, oncology and clinical nutrition literature. To ensure exhaustiveness, the reference lists of the included studies were also analyzed.

### 2.3. Study Selection

After removing duplicates, identified articles were subjected to title and abstract screening, followed by full-text assessment for eligibility, according to pre-established inclusion and exclusion criteria.

**Figure 2:**
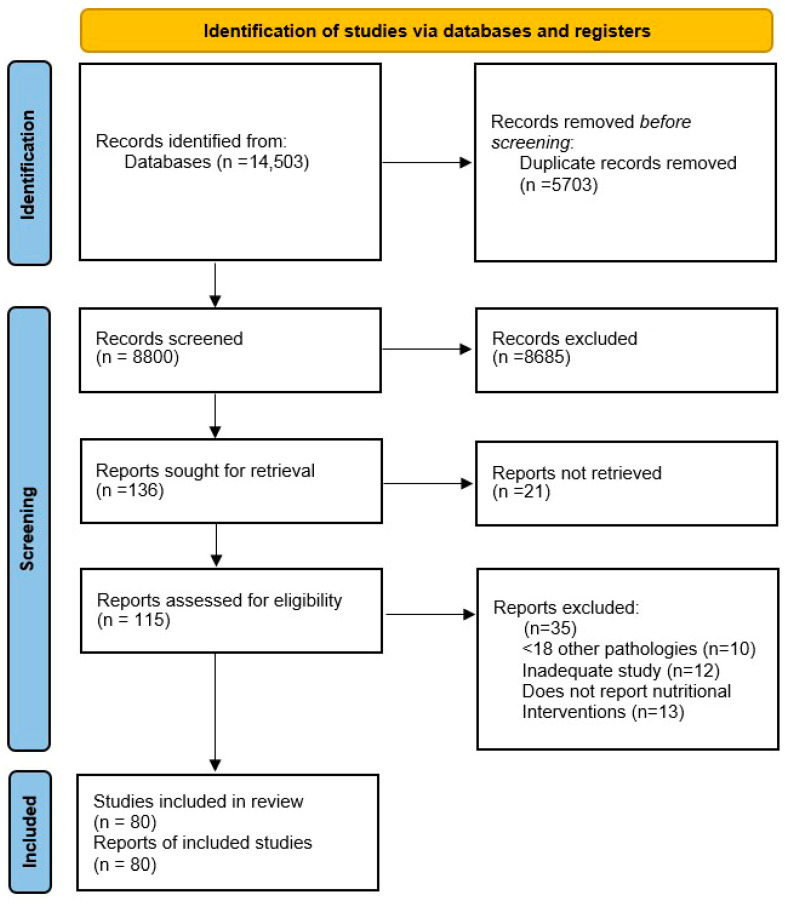
PRISMA 2020 flow chart for database and registry searches. Nutritional therapy in digestive cancer malnutrition.

The systematic search initially identified 14,503 articles. After removing 5703 duplicates, 8800 articles were assessed at title and abstract level for eligibility, of which 8685 were excluded. A total of 115 articles were assessed in full, and 35 were excluded at full-text level for inappropriate population, inappropriate study type, lack of reporting of relevant nutritional outcomes, or unavailability of full-text. Finally, 80 studies were included in the systematic review. The selection process followed the steps specified in the PRISMA 2020 flowchart presented in (Figure 2).

### 2.4. Data Extraction

Data extraction was performed by a single researcher, using a standardized data collection table and pre-established eligibility criteria, to reduce the risk of systematic errors and inconsistency. The information extracted included the following: author and year of publication, study design (cross-sectional, cohort, observational, randomized clinical trials), population characteristics (age, sex, number of participants, nationality/country, diagnosis), type of gastrointestinal cancer, methods of nutritional status assessment, monitored parameters (BMI, anthropometric measurements), type of nutritional intervention (including enteral nutrition), and main reported outcomes.

### 2.5. Data Analysis

Given the significant heterogeneity of the included studies in terms of design, populations investigated, nutritional interventions and reported outcomes, a meta-analysis was not performed. The results were summarized descriptively and qualitatively.

### 2.6. Assessment of Risk of Bias

Although a formal risk of bias assessment tool (e.g., Cochrane Risk of Bias Tool, Newcastle–Ottawa Scale, or ROBINS-I) was not applied, the methodological quality and potential sources of bias of the included studies were qualitatively assessed. This assessment took into account study design, sample size, population characteristics, type and validation of nutritional assessment instruments, and clarity and completeness of reporting of results. These elements were considered in the critical interpretation of the results and are explicitly discussed in the Section 4.

## 3. Results and Discussions

**Figure 3:**
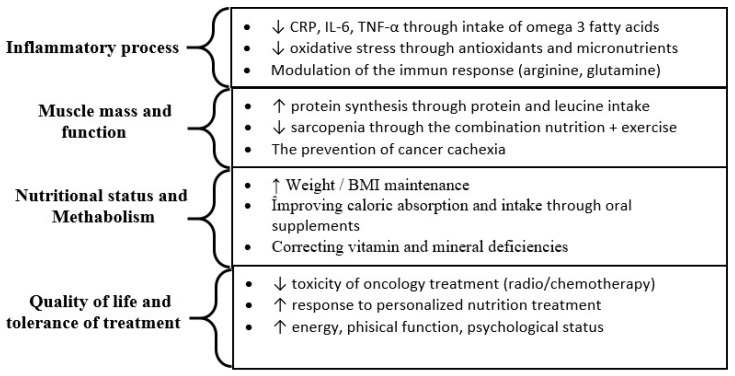
Relationship between mechanisms of nutritional interventions and impact on cancer progression. CRP = C-reactive protein, IL-6 = Interleukin 6, TNF-α = Tumor necrosis factor alpha, BMI = Body Mass Index.

This section is structured in three main parts. The first part analyzes the role of inflammation and nutritional status in the evolution of oncology patients, describing the mechanisms by which inflammatory markers (CRP, IL-6, TNF-α) and decreased BMI contribute to the development of malnutrition and cachexia. This part is also visually supported by Figure 3, which highlights the link between inflammation, muscle loss and prognosis. The second part synthesizes data from cross-sectional and cohort observational studies, which evaluated the prevalence of malnutrition, risk factors and the impact on the quality of life and survival of oncology patients. These results are presented in Table 1 and Table 2, which include both studies that emphasize the importance of early nutritional assessment and some that did not reveal a significant association. In patients with gastrointestinal cancer, advanced age is a clinically relevant factor influencing nutritional management, as older patients are at increased risk of malnutrition due to sarcopenia, comorbidities, and impaired digestive and absorptive function, requiring individualized and closer nutritional monitoring and intervention (Table 3). The third part integrates data from randomized interventional trials and meta-analyses, which evaluated the effectiveness of nutritional interventions (counseling, oral supplements, enteral and multimodal support) on the evolution of patients. These results are presented in Table 4 and Table 5 and include both positive findings (improvement in nutritional status, treatment tolerance, and survival) and negative or neutral results.

### 3.1. The Role of Inflammation and Nutritional Status in the Evolution of Oncological Patients

#### 3.1.1. The Impact of Malnutrition on Prognosis

Research has shown that nutritional support tailored to the individual patient’s needs can reduce the length of hospital stay, reduce treatment-related toxicity, and improve nutrient intake, quality of life, and physical function, thereby facilitating successful completion of cancer therapy by preventing malnutrition.

Nearly a quarter of cancer patients are at risk of death from the consequences of malnutrition rather than from the disease itself, and patients with gastrointestinal neoplasms are at increased risk of malnutrition due to the gastrointestinal tract damage caused by the underlying pathology [23]. Cancer patients often report a significant reduction in food intake after initiating therapy compared with the period before diagnosis. Some studies have shown a general decrease in food intake, while others have shown a typical reduction in protein intake. This decrease in food intake contributes to weight loss and a reduction in meal frequency.

#### 3.1.2. Systemic Inflammation and Muscle Loss

All oncological patients frequently experience a systemic inflammatory response syndrome, which can vary in intensity, and which is associated with fatigue, decreased physical capacity, anorexia and weight loss. This syndrome causes changes in carbohydrate, lipid and protein metabolism, increasing the risk of malnutrition. The reasons that lead to a reduction in food intake are multiple and include nausea, vomiting, intestinal transit disorders and anorexia, all of which contribute to weight loss.

Oncological treatment, as well as surgical treatment, can lead to a decrease in the intake of macronutrients: carbohydrates, lipids and proteins. In addition, systemic inflammation, mediated by cytokines such as IL-1, IL-6 and TNF-α produced by the tumor, causes the degradation of muscle proteins and adipose tissue, accentuating weight loss [24].

#### 3.1.3. Changes in Dietary Intake and the Effects of Oncological Therapy

The risk of malnutrition differs depending on the type of cancer, with patients with digestive tract cancers presenting a higher risk of malnutrition, influenced by the stage and progression of the disease. To maintain a stable nutritional status, the diet of the oncological patient must primarily satisfy their energy needs. Surgical interventions, performed to remove the cancerous tumor and support the oncological treatment, are often followed by insufficient food intake, which favors the occurrence of malnutrition, constituting an important risk factor for postoperative complications. Malnourished patients undergoing surgery have a higher incidence of incisional infections and reduced survival.

Studies show that patients undergoing surgery need adequate and personalized nutritional support to support recovery, and early mobilization helps maintain muscle function.

The surgical method has a direct and significant impact on the need for nutritional changes in patients with gastrointestinal cancer. Oncological digestive surgery can alter the anatomy and function of the gastrointestinal tract, affecting digestion, nutrient absorption and the ability to resume oral nutrition, which increases the risk of malnutrition and postoperative complications. For this reason, international guidelines recommend systematic preoperative nutritional assessment and adaptation of nutritional support according to the type of intervention, with priority for early resumption of oral nutrition or enteral nutrition when the gastrointestinal tract is functional. Parenteral nutrition is reserved for situations in which the use of the digestive tract is not possible or insufficient. These recommendations are supported by evidence showing that appropriate perioperative nutritional interventions reduce complications, length of hospital stay and improve postoperative recovery in patients with gastrointestinal cancer [13,25,26,27].

Since oral nutrition is not always sufficient, it is supplemented, when necessary, with enteral or parenteral nutrition, adapted to each patient according to energy needs and clinical status. To clarify how nutritional interventions influence patient outcomes, Figure 3 schematically shows the relationship between inflammation, muscle loss, and the effects of nutritional support on clinical outcomes.

### 3.2. Cross-Sectional and Cohort Observational Studies That Assessed the Prevalence of Malnutrition, Risk Factors and Survival of Oncological Patients

#### 3.2.1. Malnutrition or Poor Diet in Patients with GI Cancer

Internationally, studies show that malnutrition affects between 20% and over 70% of patients diagnosed with oncological disease. It is estimated that between 10 and 20% of deaths associated with neoplasms are caused by the consequences of malnutrition, not by the direct evolution of the disease. Although certain categories of patients are clearly more at risk, many do not receive adequate nutritional assessment and intervention. The situation is particularly critical after major gastrointestinal surgery such as gastrectomy, pancreatectomy or intestinal resections and in cases with high stomas or therapy-induced diarrhea. In such contexts, up to 80% of patients with digestive tract tumors and almost 30% of oncological patients in general lose weight even before the diagnosis is confirmed [28].

In a prospective study of 328 patients with metastatic disease or in neoadjuvant therapy, nutritional deficiencies were detected early by daily questionnaires, before the onset of clinical signs [8]. Malnutrition and cachexia were frequently clinically underestimated, and symptoms such as taste disturbances (76%), changes in smell (45%), and xerostomia highlight the need for an integrated nutritional assessment. Early detection and correction of nutritional deficiencies can significantly improve clinical outcomes [29].

Another study of 1473 cancer patients showed that muscle wasting is an important predictor of survival. The assessment included weight loss history, lumbar muscle mass and quality, measured by CT, and the results showed that these muscle changes are important prognostic factors for survival regardless of BMI [30].

Preoperative nutritional status, especially reduced muscle mass, influences postoperative outcome and long-term survival. However, the effect of muscle atrophy and malnutrition on complications, length of hospital stay and mortality remains unclear, and the limitations of existing studies emphasize the need for further research [31].

A complex syndrome characterized by progressive wasting is cancer-associated cachexia. Reduced food intake is common and is associated with significant weight loss, and many patients fail to achieve the recommended intake even with nutritional support, highlighting the difficulty of managing this syndrome [32]. A multicenter study conducted on 1545 cancer patients showed that one in three is malnourished, which is associated with longer hospitalizations, and in the case of pre-existing obesity, the detection of nutritional deficiency may be delayed and severe malnutrition significantly increases mortality [33].

Early nutritional intervention is recommended for all cancer patients at risk of malnutrition but who can maintain oral intake. This includes dietary counseling, management of symptoms that affect nutrition, and protein and energy nutritional supplements. If intake remains insufficient, enteral nutrition is resorted to, and in case of impossibility, parenteral nutrition. Such interventions increase weight and energy intake and improve quality of life, especially in patients undergoing radiotherapy [13].

Malnutrition affects quality of life, increases the toxicity of therapy and contributes to 10–20% of deaths, regardless of the evolution of the disease, therefore nutritional assessment and management are essential and should be initiated from the diagnosis [25]. Numerous studies show that in patients with colorectal cancer, dietary regimens influence the evolution of the disease: a healthy diet can prevent progression, and a poor body composition increases the risk of postoperative complications and the duration of hospitalization [34].

The indications for nutritional support in oncological patients depend on the stage of the disease and the type of treatment, so regular monitoring of nutritional status is essential. Nutritional interventions aim to optimize clinical outcomes by compensating for insufficient energy intake. Nutritional support is recommended for malnourished or at-risk patients, especially if oral intake is <60% of the required for more than 7 days. The first step consists of dietary counseling and oral nutritional supplements, and if intake remains insufficient and intestinal function is normal, enteral nutrition is recommended, and in case of impossibility, parenteral nutrition [35]. If cachexia sets in, it manifests itself through progressive weight loss, reduced muscle mass and an increasingly low food intake, or with pre-existing sarcopenia, which worsens the clinical course and limits therapeutic options [36].

Recent data on body composition indicate that the association between excess fat and low muscle mass, also called sarcopenic obesity, may increase the risk of complications and mortality in non-malignant diseases and may amplify the toxicity of chemotherapy [37]. To provide a clear picture of the link between nutritional status and prognosis in cancer patients, Table 1 summarizes the main cross-sectional and observational studies dedicated to this topic. These studies consistently show that nutritional parameters influence clinical outcome in various types of cancer, highlighting the importance of nutritional assessment as an integrated part of cancer care.

**Table 1:** Cross-sectional and observational studies on nutritional status and prognosis in oncology patients.

First Author/Year	Type of Study	Study Population (N)	Cancer Type	Nutritional Parameter Evaluated	Main Results	Ref.
Nakazono, 2021	Comparative observational Study	150	Cancer Gastric	Post-gastrectomy food intake	Total gastrectomy is associated with significantly greater food losses compared to other types of gastric resections, having direct implications on postoperative nutritional status.	[5]
Rasschaert, 2024	Prospective observational cross-sectional	328	Diverse Cancers	Malnutrition screening	Prevalence of malnutrition and cachexia.	[8]
Amezaga J, 2018	Transverse	151	Various cancers	Assessment of self-reported chemosensory changes in patients undergoing chemotherapy	Changes in taste and smell are common side effects in cancer patients undergoing chemotherapy treatments.	[29]
Durán Poveda, 2023	Prospective, observational, multicenter	469	Cancer GI	MUST, PG-SGA	40% of patients are at risk of malnutrition and increased mortality.	[19]
Pressoir, 2010	Cross-sectional observational, prospective multicenter	1545	Various cancers (predominantly digestive)	IMC	Prevalence of malnutrition, correlation with length of hospitalization and mortality.	[33]
Hébuterne, 2014	Transverse	1903	Digestive Cancers	IMC, weight loss	39% of patients present with malnutrition.	[38]
Prado, 2008	Transverse	2115	Digestive/GI cancers	Muscle mass (CT)	Sarcopenia is associated with reduced survival.	[37]
de Pinho, 2019	Transverse	4783	Various cancers	Increased risk of malnutrition	Prevalence and risk factors of malnutrition in hospitalized cancer patients.	[39]
Flynn, 2018	Transverse	140	Various cancers	Implementation of nutritional screening tools	Managing cancer cachexia	[40]

GI = Gastrointestinal; CT = Computed tomography; MUST = Malnutrition universal screening Tool; PG-SGA = Patient generated subjective global assessment.

Existing studies have examined the relationship between muscle loss during chemotherapy and the prognosis of patients with inoperable colorectal cancer. Although reduced muscle mass before treatment did not influence survival, a significant decrease in muscle mass after chemotherapy (>5%) was associated with a lower overall and progression-free survival [37,41]. Several studies have shown that muscle atrophy is consistently associated with a poorer prognosis in patients with different types of neoplasms. At the same time, sarcopenia seems to increase the risk of toxicity of oncological treatments.

However, prospective studies are needed to clarify whether monitoring and intervention on muscle mass can bring real clinical benefits in this category of patients [41]. Studies show that, in colon cancer, laparoscopic surgery allows for faster recovery and shorter hospitalization compared to classical surgery. However, patients with sarcopenia are at higher risk of complications after surgery, regardless of the technique used. Laparoscopic surgery can partially reduce the negative impact of sarcopenia on short-term postoperative outcomes [42].

A study in a cohort of patients with advanced disease showed that low muscle mass is associated with reduced physical functioning, independent of age or stage of disease. In men, lower muscle level was additionally correlated with increased fatigue and poorer quality of life. The accumulation of adipose tissue in muscles was associated with dyspnea and functional limitations in both sexes, but after taking into account other factors, only the link with fatigue was associated in men [43].

In patients with esophageal, pancreaticobiliary, or lung cancer, compared with those with gastric, hepatic, or colorectal neoplasms, malnutrition is more common and more severe. The risk is particularly increased in patients undergoing chemotherapy or radiotherapy, compared with those treated surgically alone. This problem has important consequences, affecting both survival and quality of life [44]. Although sarcopenia and sarcopenic obesity do not appear to directly affect liver function, obese patients often have an enlarged liver with reduced function, showing that size does not reflect liver performance [30].

Almost half of cancer patients develop cachexia, with progressive loss of muscle and fat mass, weight loss, and reduced quality of life. This involves increased energy expenditure and muscle protein degradation, stimulated by tumor and inflammatory factors. Although the mechanisms are better understood, effective therapies remain limited [45].

Cohort studies confirm the data obtained from cross-sectional studies, providing a longitudinal perspective on the relationship between nutritional status and clinical outcome of oncology patients. Table 2 summarizes the main relevant studies, highlighting the nutritional impact on prognosis and survival, underlining the importance of nutritional assessment in oncology management.

**Table 2:** Cohort studies on the association of nutritional status with the prognosis of oncological patients.

First Author, Year	Type of Study	Population (N)	Cancer Type	Follow-Up Period	Nutritional Parameter Evaluated	Main Results	Ref.
Miyamoto, 2015	Observational cohort	215	Inoperable CCR	8 years	Weight loss, skeletal muscle analysis	Loss of muscle mass has a negative prognosis.	[41]
Oh, R.K, 2020	Prospective cohort	423	Colorectal	4 years	Muscle mass, CT scan	Sarcopenia has been associated with postoperative complications after laparoscopic surgery for colon cancer.	[42]
Boulahssass, 2019	Observational cohort	3140	Various digestive cancers	6 years	Malnutrition status	Poor diets correlated with decreased survival.	[44]
Choi M.H, 2018	Retrospective cohort	188	Advanced rectal cancer	52 months	Nutritional status, sarcopenia	Sarcopenia associated with decreased survival.	[46]
Fettig A, 2025	Observational cohort	2000	Various types of cancer	10 years	Adverse effects of treatment, nutrition combined with exercise and relaxation	Patients demand more personalized information on diet and nutritional support, analysis suggests communication gaps.	[47]
Martin, 2015	Observational cohort	8160	Various types of cancer	2 years	Causes of death	Weight loss is unclear.	[48]
Feriolli, 2012	Observational cohort, prospective	162	Operable gastrointestinal cancer	5–6 weeks after surgery	Monitoring physical activity at different stages	Physical activity correlates with disease stage and quality of life.	[49]
Bozzetti, 2014	Observational cohort	414	Incurable cachexia	6 years	Parenteral nutrition	The role of parenteral nutrition is controversial.	[50]
Velasquez, 2023	Observational cohort	68	Various types of cancer	6 months	Nutritional assessment	Complications of parenteral nutrition.	[51]
Jeannine Bachmann, 2008	Observational cohort	198	Pancreatic cancer	18 months	Massive loss of adipose tissue	Due to skeletal muscle loss, many cachexia patients develop pulmonary failure with dyspnea as a common symptom.	[52]

CCR = Colorectal cancer; CT scan = Computed tomography scan.

#### 3.2.2. The Relationship Between Nutritional Intervention and Patient Age

The age of the patient with gastrointestinal cancer is a significant parameter in how nutritional intervention is adapted. These are not completely different rules, but individualized adaptations based on physiological needs and age-specific risks. In elderly patients, there is a physiological loss of muscle mass, which can be aggravated in the context of GI cancer and malnutrition [53,54]. This increases the need for high-quality protein and adequate caloric intake to maintain muscle mass.

Also, in older people, there may be comorbidities and physiological functions; they may have associated diseases (diabetes, renal failure, liver failure) that influence nutrient absorption and tolerance to certain foods or supplements [25]. Altered digestion and absorption (decreased gastric secretion, slower intestinal motility) may affect tolerance to certain foods, the need for frequent meals, and supplementation with enzymes or essential nutrients [45]. There are studies showing that age over 65 years is an independent risk factor for malnutrition in patients with GI cancer [55].

This implies more frequent monitoring and proactive nutritional interventions (oral supplements, texture adaptations, enteral nutrition if necessary) (Table 3).

**Table 3:** Proactive nutritional interventions by age.

Appearance	Young Patients	Elderly Patients	References
Protein intake	1.2–1.5 g/kg/day	1.5 g/kg/day or more to prevent sarcopenia	[54,55]
Calories	Adequate according to weight and activity	Adequate, more careful monitoring for rapid weight loss	[25,55]
Supplements	ONS if dietary intake insufficient	ONS frequently recommended, possibly with anti-inflammatory nutrients (e.g., omega 3)	[25,45]
Food texture/consistency	Normal	Possibly adapted for dysphagia, dental problems or reduced digestion	[25]
Monitoring	Standard	More frequent: weight, muscle mass, nutritional laboratory	[25,54,55]

ONS—Oral Nutritional Supplements.

In the elderly, unintentional weight loss, which is very common, can promote frailty, cachexia and infectious processes, thus increasing the risk of unfavorable evolution and premature mortality. In overweight patients, this sign may be underestimated, and untreated malnutrition may progress, requiring medical intervention [38].

#### 3.2.3. Malnutrition and Nutritional Management in Patients with GI Cancer

It has been observed that advanced stage of GI cancer (stages III-IV or metastatic) is frequently associated with malnutrition and cachexia. As the disease progresses, patients may experience decreased appetite, digestive difficulties, and tumor-induced systemic inflammation, which increases the risk of significant nutritional deficiencies [56,57]. Nutritional management should be personalized and multidisciplinary. Thus, it should include caloric and protein supplementation (hypercaloric and hyperprotein diet, adapted to digestive tolerance), oral protein and calorie supplements (shakes, protein powders) if normal food intake is insufficient [56,58].

Specific supplements (omega-3 fatty acids, to reduce inflammation associated with cachexia) [56] and vitamins and minerals (B, D, zinc) in case of documented deficiencies [57,59] would also be necessary. In addition, dietary adjustments should be made, such as easily digestible foods, frequent and small meals [58] and avoidance of foods that aggravate digestive symptoms, correction of taste disorders and xerostomia [56,57]. All this management should be carried out through specialized support, consisting of clinical nutritionists and interdisciplinary teams, to monitor weight and protein requirements, and in severe cases, if necessary, by administering enteral or parenteral nutrition [56,57,58].

Nutritional interventions can reduce gastrointestinal symptoms and improve tolerance to oncological treatments for patients with digestive tumors, regardless of whether the disease is metastatic or not. Personalized nutritional interventions are made according to digestive symptoms and absorptive capacity. Thus, patients with nausea, vomiting or diarrhea may require specialized dietary counseling, patients with malabsorption or dysgeusia may require nutritional supplements or adaptations of dietary texture/composition, and patients with rapid weight loss or cachexia may require more aggressive nutritional support (enteral/parenteral) [56].

#### 3.2.4. Postoperative and Posttreatment Nutritional Management in Patients with GI Cancer

Postoperative and posttreatment nutritional support plays an important role in the management of nutritional interventions in patients with gastrointestinal cancer. It is an essential component in the management of patients with gastrointestinal cancer, having a direct impact on recovery, tolerance to oncological treatments and prognosis. After surgery and/or systemic treatments (chemotherapy, radiotherapy), patients frequently present with reduced food intake, malabsorption, weight loss and sarcopenia, which justifies the need for structured and individually adapted nutritional interventions [13,26].

According to ESPEN guidelines, early resumption of oral nutrition is recommended as soon as it is tolerated, as it maintains intestinal function, reduces infectious complications and shortens the length of hospital stay [25,26]. When oral intake is insufficient, oral nutritional supplementation (ONS) is the first line of intervention, and enteral nutrition is indicated if nutritional needs cannot be met by oral nutrition [13,25]. Parenteral nutrition is reserved exclusively for situations in which the gastrointestinal tract cannot be used or is insufficiently functional, given the increased risk of metabolic and infectious complications [26,60].

In the post-cancer treatment period, nutritional support aims to prevent and correct malnutrition, maintain muscle mass, and alleviate persistent digestive symptoms (dysgeusia, diarrhea, early satiety). Continuous nutritional counseling, periodic monitoring of nutritional status, and the use of high-protein and high-calorie supplements are recommended, especially in elderly patients or patients with advanced disease [13,36]. Evidence suggests that these strategies contribute to improving quality of life, treatment tolerance, and reducing postoperative complications and readmissions [13,25,26,60].

Randomized interventional studies and meta-analyses that evaluated the effectiveness of nutritional interventions on patient outcomes.

A meta-analysis of 27 studies showed that patients with sarcopenia have a higher risk of complications at 30 days postoperatively. These data confirm that sarcopenia influences postoperative outcome in gastrointestinal neoplasms, especially colorectal cancer, and emphasize the importance of recognizing and managing it as a preoperative prognostic marker [61]. Clinical outcomes are affected by the side effects of chemotherapy, and exercise reduces fatigue, supports psychological well-being, and may improve physical function without clearly influencing dose intensity. To explore this issue, the National Cancer Institute has funded the ENICTO Consortium, which is studying the role of exercise and nutrition in increasing treatment tolerance and improving oncological outcomes, with the goal of integrating these interventions as a complementary standard of care to chemotherapy [62].

A randomized controlled trial showed that individualized nutritional intervention, tailored to symptoms, improves chemotherapy tolerance, nutritional status, and quality of life, including physical function, in postoperative colorectal cancer patients [63]. In the case of oral cancers, extensive surgery affects nutritional status and increases the risk of complications. Personalized postoperative nutritional support reduced weight loss, maintained muscle function and micronutrient levels, and improved physical activity and eating habits. These data emphasize the importance of early screening and individualized nutrition [64].

Adequate nutrition is crucial for cancer patients, but the optimal timing and mode of intervention are still not clearly established. A 2022 National Institutes of Health workshop showed that existing studies are heterogeneous and of variable quality, although some indicate benefits of nutritional interventions in reducing adverse effects. Experts recommend systematic nutritional screening and referral of patients at risk to dietitians, emphasizing the need for rigorous studies on the effectiveness and cost-effectiveness of these strategies [65].

A study of 54 patients showed that early nutritional intervention, started at the same time as radiotherapy, reduces weight loss and the incidence of severe oral side effects, improving nutritional parameters and PG-SGA scores compared with late intervention [66].

International guidelines recommend oral nutritional interventions for malnourished or at-risk patients, which increase food intake and improve certain aspects of quality of life, without influencing mortality [67]. A randomized clinical trial on 159 patients undergoing radiotherapy showed that oral nutritional supplements, along with nutritional counseling, reduce the negative effects of radiotherapy, improving quality of life and tolerance to treatment compared to counseling alone [68].

A randomized trial showed that a psychologically supported nutritional program, implemented by oncology nutritionists, improves nutritional status, reduces weight loss and treatment interruptions, improves depression, and increases quality of life, supporting the integration of this approach into standard care [69].

A randomized trial showed that home enteral nutrition, compared with nutritional counseling, helps patients with upper GI cancer and increased nutritional risk to maintain their weight and complete chemotherapy as planned, being a feasible, safe intervention with no negative impact on quality of life [70]. Cachexia, characterized by weight loss, anorexia, and functional decline, requires multimodal interventions. Following a phase II clinical trial with favorable results, a phase III randomized clinical trial evaluating this multimodal intervention is underway [16]. The evidence from previous studies is supported and complemented by the randomized controlled trials (RCTs) in Table 4, which provide an assessment of the causal effect of nutritional interventions.

**Table 4:** Randomized controlled trials (RCTs) on the effectiveness of nutritional interventions.

First Author, Year	Type of Study	Population (N)	Cancer Type	Intervention	Follow-Up Period	Main Results	Ref.
Nett et al., 2022	RCT	62	Diverse	Oral supplement + physical activity + personalized nutrition	6 months	Improving nutritional status and quality of life	[64]
Gavazzi, 2016	RCT	79	Upper GI, malnutrition	Home enteral nutrition vs. control	3 months	Home enteral nutrition improved weight and quality of life	[70]
Wang et al., 2025	RCT	88	Colorectal	Individualized nutrition postoperative	12 months	Improving both chemotherapy tolerance and quality of life	[63]
Basch, 2017	RCT	766	Various types of cancer	Reported parameters, nutritional intervention	4 ani	Results on the link between reported symptoms and survival	[71]
Ryan, 2009	RCT	53	Esophagus	Enteral nutrition+ EPA	5 days preoperative, 21 days postoperative	Early supplementation of enteral nutrition (EN) with eicosapentaenoic acid (EPA) was associated with superior preservation of lean muscle mass post esophagectomy compared with standard enteral nutrition	[72]

GI—gastrointestinal cancer, EN—enteral nutrition, EPA—eicosapentaenoic acid.

The overall 5-year survival rate for all cancers is 68%, with over 16.9 million survivors in the United States. Evidence suggests that diet, physical activity, and obesity influence the risk of recurrence and survival. The American Cancer Society guidelines provide evidence-based recommendations on nutrition, physical activity, alcohol consumption, and anthropometric parameters, addressed to patients, physicians, and families, and include guidance for interventions during treatment and post-treatment support [73]. The European MyPath project brings nutritional assessment and intervention into the oncology routine, putting the patient at the center. By using standardized digital tools and patient-reported data, the system provides personalized nutritional recommendations and ongoing monitoring, so that interventions are constantly adapted to the needs of each patient [74].

A systematic review suggests that enteral nutrition, especially when guided by dietitians or combined with parenteral nutrition, may improve the quality of life of cancer patients, although further studies are needed due to the limited number and heterogeneity of existing studies [75]. Cancer cachexia is recognized as being closely related to systemic inflammation. Thus, patients at risk are identified by combining it with decreased food intake and inflammatory activation, providing a clearer framework for diagnosis, prognosis and multimodal therapeutic strategies [76]. Comprehensive oncology care involves evidence-based personalized nutrition, integrated early, to improve clinical outcomes, quality of life and patient well-being, while being both an ethical obligation and a cost-effective strategy [77].

In elderly patients diagnosed with esophageal carcinoma after surgery, enteral nutrition improves nutritional status (BMI), serum parameters, nutritional risk without increasing complications, and supports nutritional recovery in the first months postoperatively [78]. In colorectal cancer survivors, healthy lifestyle behaviors are influenced by individual, psychological, and social factors, being inhibited by smoking, pain, and depression, but supported by motivation, good functional status, knowledge, and social support [79]. In patients with upper gastrointestinal cancer, personalized nutrition, combined with physical activity, may improve physical function, although the effects on muscle mass and quality of life are still unclear. Given the high prevalence of malnutrition and the negative impact on disease progression, guidelines recommend preoperative nutritional support (1–2 weeks), preferably by oral supplementation, but evidence regarding clinical benefits remains inconsistent [58,80].

Systematic reviews and meta-analyses have examined associations between foods, beverages, nutrients, and the risk of colorectal cancer (CRC), but comprehensive assessments of the quality of this evidence remain limited [81].

Nutritional interventions in adults with cancer have limited evidence due to small and varied studies, but sarcopenia, severe loss of muscle mass, is associated with poor survival in metastatic colorectal cancer [10,82]. Table 5 summarizes reviews and meta-analyses that show the effectiveness of nutritional interventions and highlights the importance of a personalized approach in the care of patients with gastrointestinal cancer.

**Table 5:** Review/meta-analysis studies on nutritional interventions in patients with GI cancer.

First Author, Year	Type of Study	Number of Studies Included	Type of Cancer	Analyzed Intervention	The Main Conclusion	Ref.
Minhajat, 2023	Systematic review	1424	Colorectal Cancer	Diet and nutritional status, the impact of BMI	Nutritional deficiency is associated with a poor prognosis.	[34]
de Vries-ten Have, 2025	Systematic review	21	CRC survivors	Determinants of healthy behaviors (diet, activity)	Identifies barriers and facilitators for adopting a healthy lifestyle in CRC survivors.	[79]
Sadeghi, 2021	Systematic review	5	Upper GI cancers	Nutrition interventions + exercises, movement	Combined interventions show benefits on nutritional status and some functional elements, but RCT data are limited and heterogeneous.	[58]
Zou, Q., 2024	Meta-analysis	12	Gastrointestinal cancers (surgery)	Perioperative nutritional support	Perioperative support improves some postoperative outcomes; varies by outcome.	[80]
Abebe, 2025	Systematic review	28	Gastro-digestive cancers	Dietary patterns by principal component analysis and reduced rank regression	Certain dietary patterns are associated with different risk/mortality; moderate conclusions.	[24]
Bouras, 2022	Umbrella review	49	Gastric cancer	Umbrella analysis	Mixed results; some consistent evidence for certain dietary exposures.	[83]
Moazzen, 2021	Systematic review and meta-analysis	44	Colorectal cancer	The role of diet quality on colorectal cancer risk	It would be recommended that general dietary advice could be provided in clinical settings.	[84]
Veettil, 2021	Review and meta-analysis	45	Colorectal cancer	Dietary plan rich in protein, fiber	Synthesizes evidence and degree of consistency; some robust associations.	[85]
Cortés-Aguilar, 2024	Review and meta-analysis	21	Metastatic colorectal cancer	Screening tools	MUST is a precision instrument.	[82]
Spei, 2023	Systematic review and meta-analysis	19	Cancer survivors	Dietary pattern, post-diagnosis	Certain post-diagnosis patterns associated with mortality; moderate evidence.	[86]
Sealy MJ, 2016	Systematic review	160	Cancer patients	Malnutrition evaluation	Thirty-seven methods for assessing malnutrition were identified, but none have acceptable content validity, compared to a construct based on the ESPEN and ASPEN definitions of malnutrition.	[87]
Keshavjee S., 2025	Systematic review	27	Post-operative colorectal cancer	The impact of sarcopenia on post-surgical condition	Preoperative sarcopenia increases the risk of complications, length of hospital stay, and postoperative mortality.	[61]
Tsilidis, 2024	Systematic review	124	Post-diagnosis colorectal cancer	Obesity, physical activity, diet, supplements	Moderately vigorous physical activity and a healthy diet are correlated with improved prognosis; adiposity and sedentary behavior correlate with a poor prognosis.	[88]
Chan, 2024	Systematic review	69	Colorectal cancer	Post-diagnosis dietary factors and supplements	Diets high in fiber, whole grains, fruits/vegetables, and moderate dairy intake are associated with better survival; red meat, alcohol, and Ca/Vit D supplements show mixed evidence.	[89]
Fretwell, 2025	Systematic review	28	Colorectal cancer	The role of diet after diagnosis	Evidence on post-diagnosis diet remains insufficient; positive trends for Mediterranean-type and high-fiber diets.	[90]
Aya V, 2021	Systematic review	17	Active patients	Physical activity and gut microbiota	Studies show subtle changes in the diversity and abundance of certain bacteria in active individuals; recommendations for more standardized measurements.	[91]
Dewiasty, 2024	Systematic review and meta-analysis	15	Institutionalized elderly people in Indonesia	Prevalence of malnutrition, nutritional intake	The prevalence of malnutrition varies widely: 6.5–48.3% (hospitals) and 3.2–61.0% (other institutions); frequent protein, Ca, Vit D deficiencies were highlighted.	[92]
Hosseini, 2025	Systematic review and meta-analysis	19	Cancer patients (various)	Prevalence of severe malnutrition	Severe malnutrition.	[93]
Inciong, 2020	Systematic review	92	Hospitalized patients in Northeast and Southeast Asia	Prevalence of malnutrition, consequences	Poor nutritional status is associated with increased morbidity and mortality and increased healthcare costs. Further research is needed.	[94]
Keesari, 2024	Systematic review	8	Patients with prediabetes and increased risk of colorectal cancer	Associated metabolic risk	The probability of developing CRC is 16% higher in patients with prediabetes.	[95]

MUST = Malnutrition universal screening Tool.

Meta-analyses have compared four malnutrition screening tools with the SGA (Subjective Global Assessment) and (European Society for Clinical Nutrition and Metabolism) standards, and MUST (Malnutrition Universal Screening Tool) has shown the highest accuracy in hospitalized adults, although variable study quality may influence the results [39].

Cancer-associated malnutrition is an independent risk factor for adverse clinical outcomes [46]. Sarcopenia is associated with reduced overall survival in patients with advanced rectal cancer, and visceral obesity tends to decrease disease-free survival.

### 3.3. Dietary Patterns Related to Prevention and Survival

#### 3.3.1. Dietary Patterns and GI Cancer Prevention

Healthy diets characterized by high consumption of fruits, vegetables, whole grains and plant-based foods are associated with a reduced risk of gastrointestinal cancer. There are meta-analyses showing that “healthy” (prudent/plant-based) dietary patterns are associated with a lower risk of gastric and colorectal cancer, while “Western/unhealthy” (high in processed meat, fat and sugar) patterns are associated with an increased risk of these cancers [96]. Examples of “healthy” diets include the Mediterranean, DASH, or those rich in fiber and antioxidants, which reduce the risk of gastrointestinal cancer through anti-inflammatory and antioxidant mechanisms [97].

#### 3.3.2. Dietary Patterns for Survival in Patients with GI Cancer

Evidence suggests that, in cancer survivors, healthy dietary patterns rich in plant-based foods are associated with reduced overall mortality and risk of gastrointestinal cancers, while Western-style diets increase this risk; however, the impact on cancer-specific mortality remains unclear and requires further study [24,86].

There are studies that show that certain dietary patterns are associated with both the prevention of gastrointestinal cancers and patient survival after diagnosis. Epidemiological studies and meta-analyses indicate that Mediterranean-type dietary patterns, characterized by high intake of fruits, vegetables, whole grains, fish, and unsaturated fats, are associated with a reduced risk of colorectal and gastric cancers, as well as with lower mortality after diagnosis [98,99,100].

Regarding survival, observational data suggest that adherence to a healthy dietary pattern after diagnosis of gastrointestinal cancer is associated with better overall survival and quality of life, likely by reducing inflammation, maintaining muscle mass, and improving tolerance to cancer treatments [100,101]. In contrast, Western dietary patterns, rich in ultra-processed foods, red and processed meats, and refined sugars, are associated with an increased risk of colorectal cancer and worse prognostic outcomes [98,102].

However, clinical guidelines emphasize that, in the active phases of cancer treatment or in advanced disease, the nutritional priority is the prevention and correction of malnutrition, and dietary recommendations should be individually adapted, even if this temporarily implies a departure from an ideal dietary pattern from a preventive point of view [13]. Thus, healthy dietary patterns have an important role in prevention and long-term survival, but they must be flexibly integrated into the clinical context of each patient.

An umbrella analysis of meta-analyses shows that obesity, increased waist circumference, alcohol and salted fish increase the risk of gastric cancers, while a healthy lifestyle and normal weight have protective effects. Adopting balanced dietary habits and reducing modifiable risk factors are important strategies for prevention [83].

A high-quality diet based on a Mediterranean diet is associated with improved cancer survival and reduced mortality. Meta-analyses show a 23% reduction in overall mortality in cancer survivors.

Further research is needed and dietary recommendations need to be adapted to effectively prevent colorectal cancer, taking into account diet, lifestyle, ethnicity and geographical region [21,84]. A healthy diet, such as the Mediterranean diet, the DASH (Dietary Approaches to Stop Hypertension) diet or the anti-inflammatory diet, tends to reduce the risk of colorectal cancer. However, the evidence is still weak and variable, making it difficult to formulate clear recommendations [85].

Future research should test screening tools in well-defined populations and optimize patient status according to body composition [88].

The impact of modifiable lifestyle factors after diagnosis may contribute to the development of personalized prevention and management strategies for colorectal cancer survivors [89]. Lifestyle changes are effective prevention strategies that can contribute to improving the survival of cancer patients [47].

Although diet influences the risk of colorectal cancer, the impact of diet on disease-specific mortality and survival remains poorly understood [103]. Integrating nutritional therapy into palliative care as early as possible improves the quality of life of oncological patients and can prolong survival, while having much lower costs than new drug therapies [104]. Immunonutrition administered to patients with upper gastrointestinal cancers, perioperatively significantly reduces infectious complications, and decreases their risk by approximately 30% [105]. Obese but malnourished patients have a much worse prognosis, which requires that weight loss recommendations be made with caution and primarily oriented towards nutritional optimization [91].

A detailed assessment of diet, sleep and circadian rhythm, of the entire lifestyle is essential, to better understand how they influence oncological evolution and prognosis. Larger and better controlled clinical trials are needed, including different types of exercise (from endurance to high-intensity training), depending on tolerance in different age groups, and integrated approaches [106].

Malnutrition impairs immunity and anabolic processes, disrupting tissue regeneration, which increases the risk of postoperative complications and perpetuates a vicious cycle between infection and malnutrition [92].

Although hospital nutritional guidelines are well established, the lack of national recommendations for nursing homes is concerning. Residents with chronic diseases and complex nutritional needs have a high prevalence of malnutrition and inadequate nutrient intake, highlighting the need for standardized guidelines for nutrition and hydration in these facilities [77]. Although healthcare is becoming increasingly patient-centered, access to evidence-based nutritional interventions is limited, hindering the achievement of this goal.

Malnutrition negatively impacts clinical outcomes and quality of life, but the benefits of early nutritional interventions are still insufficiently recognized, and overall evidence remains limited due to the small number of available studies [75,107]. Simple and reliable techniques, such as bioelectrical impedance analysis (BIA) and ultrasound, allow early and individualized diagnosis of malnutrition at the beginning of radiotherapy and should be used in clinical routine to identify patients who require intensified and personalized nutritional support [93]. Continuous monitoring of nutritional status and associated socioeconomic factors is an essential element during treatment [94].

A systematic review shows that hospital malnutrition is more common in Northeast and Southeast Asia than in Europe and Latin America, and is associated with increased morbidity, mortality, and costs, in a context of low awareness. Further research is needed to improve the identification of patients at nutritional risk [108].

Nutritional support is essential for optimizing postoperative outcomes in colorectal cancer. Early outpatient nutritional assessment allows for the identification of patients at risk and allows for the implementation of interventions in the preoperative period to improve the general condition and postoperative prognosis [95].

A study shows that patients with prediabetes have a higher risk of colorectal cancer compared to those with normal blood glucose, emphasizing the importance of lifestyle modification and screening in high-risk individuals [109]. The Global Burden of Diseases 2019 study provided detailed estimates of cancer incidence, morbidity, and mortality, supporting local and global efforts to reduce this disease [110].

An effective diet for preventing the adverse effects of chemotherapy in patients with esophageal cancer is unclear. Although it did not significantly reduce gastrointestinal toxicity, the diet significantly reduced the risk of neutropenia and leukopenia, and had a protective effect on hematological toxicity [111]. In a large cohort of young patients with hematological malignancies such as leukemias and lymphomas, who are at risk of non-cancer deaths, it was observed that deaths occurred, probably as a result of treatment, in the first year after diagnosis [112].

A retrospective analysis assessed the quality of life in patients with advanced cancer treated for cachexia in a multidisciplinary clinic, identifying clinical factors associated with improved QoL, and a large prospective study demonstrated the relationship between BMI, muscle mass and overall survival, proposing a prognostic classification system [48,113]. The goal of patient-centered oncology care is to optimize survival and improve health-related quality of life [71]. Symptoms are common in patients with advanced and often undetected cancers. Their systematic collection through standardized, patient-completed questionnaires can improve symptom control [114].

Effective management of metabolic malnutrition involves maintaining a favorable balance between anabolism and catabolism, essential for muscle mass, immune function, and quality of life; given the variability of energy expenditure in oncological patients, their correct assessment and monitoring of physical activity are important for optimizing nutritional therapy [49,115,116].

A systematic review suggests that prophylactic placement of percutaneous endoscopic gastrostomy may reduce malnutrition and improve quality of life in high-risk patients [50].

### 3.4. Methods of Nutrient Administration

The method of nutrient administration is important in patients with gastrointestinal cancer. Oral administration is preferred because it maintains the integrity of the gastrointestinal tract and intestinal flora [13], is less invasive and safer compared to parenteral administration, and is the one that patients can use at home, supporting the continuity of nutritional intake [25]. Another route of nutrient administration is enteral, via tube, which is used if the patient cannot ingest enough orally (due to dysphagia, tumor obstruction, severe nausea, diarrhea, or massive weight loss), enteral nutrition via tube is recommended. Thus, there is a reduction in infectious complications and maintenance of muscle mass compared to total parenteral nutrition [117]. Parenteral (intravenous) nutrition is indicated only when the gastrointestinal tract cannot be used (e.g., complete obstruction, digestive fistulas, paralytic ileus). This route of administration, although it can support caloric and protein intake, increases the risk of complications (infections, electrolyte imbalances, hyperglycemia) [36,117].

Understanding the impact and patient experiences of PN is essential for both healthcare professionals and patients [51]. Early enteral nutrition supplementation helps maintain muscle mass even after esophagectomy and is worth studying in the long term for its impact on recovery and quality of life [72]. A systematic review shows that enteral nutritional support increases food intake and reduces postoperative complications. The benefits of EPA supplementation remain unclear, highlighting the need for further studies to establish clinical efficacy in cancer patients [118]. One study showed that adherence to the DASH (Dietary Approaches to Stop Hypertension) diet is associated with a lower risk of cancer mortality, while scores related to recommended foods or dietary diversity showed no significant correlations [119]. In cancer survivors, a high-quality diet and a prudent dietary pattern are associated with lower overall mortality, while a Western dietary pattern increases the risk of death [120]. Although high fruit and vegetable consumption may prevent cancer, studies have not consistently demonstrated a clear reduction in overall cancer risk [121].

Gastric cancer is frequently diagnosed and the second leading cause of cancer mortality in Korea, and metabolic syndrome appears to influence patient prognosis, increasing the risk of gastric dysplasia and affecting survival and tumor recurrence, highlighting the role of lifestyle interventions [122].

Nutritional symptoms are common even 12 months after chemotherapy, affecting quality of life and functional status, highlighting the importance of early identification and management [123]. Symptoms such as anorexia, dysphagia and oral ulcers present before treatment affect food intake, weight and function of patients, accelerating weight loss and negatively impacting survival [124].

In patients with advanced cancer, changes in taste and smell, dysphagia and constipation are common and often underdiagnosed, and early identification and treatment may prevent their contribution to cachexia [125]. Patients with cachexia experience more severe symptoms and have greater food discomfort compared with those without cachexia [126].

A retrospective study in colorectal cancer patients showed that approximately 40% develop cachexia within the first 12 weeks of chemotherapy, highlighting the need for early identification for multimodal interventions; the Modified Glasgow Prognostic Score (mGPS), based on CRP and albumin, can serve as an indicator of inflammation and nutritional status [127].

There are many definitions of malnutrition, generating confusion, and recent evidence points to acute or chronic inflammation as a determining factor in the pathophysiology of disease-associated malnutrition [128]. The Global Leadership Initiative on Malnutrition (GLIM) initiative proposed an international consensus for the diagnosis of malnutrition in adults, using a two-step approach: screening and assessment for diagnosis and severity. Diagnosis requires at least one phenotypic criterion (weight loss, low BMI, reduced muscle mass) and one etiological criterion (reduced food intake or inflammation) [129]. Hand grip strength is a simple, noninvasive marker with prognostic value for mortality, morbidity and recovery, indicating the risk of complications, prolonged hospitalization and loss of independence, and is also a sensitive indicator of nutritional status [130].

Pancreatic cancer, the fourth leading cause of cancer death in Western countries, is often associated with cachexia, present in ~40% of patients at the time of surgery and correlated with malnutrition, increased metastasis and reduced survival, independent of tumor size, highlighting the role of metastatic dedifferentiation [52].

A review of 160 studies on the assessment of malnutrition in cancer patients showed that no method achieved content validity according to the ESPEN and ASPEN (American Society for Parenteral and Enteral Nutrition) definitions, highlighting the need for standardization of instruments [87].

### 3.5. General Observations of the Analysis

Early and especially individualized nutritional interventions have beneficial effects on weight maintenance, muscle mass and quality of life. Reduced food intake and systemic inflammation are the main mechanisms that determine malnutrition. Malnourished patients undergoing surgery are at increased risk of postoperative complications and high mortality, which underlines the importance of nutritional support.

### 3.6. Recommendations for Future Research

Future research should include nutritional follow-up of patients, which should be early and continuous, and the establishment of individualized nutritional support to prevent malnutrition and maintain muscle function.

Future research should also focus on integrated models of nutritional interventions and mobilization from diagnosis, as well as an assessment of the impact on survival and quality of life.

### 3.7. Strengths of This Systematic Review

This subsection reviews studies that assessed how dietary interventions influence nutritional status in patients with digestive cancers. The results are not uniform: some studies show benefits, while others do not show significant effects. The review includes both cross-sectional studies, which examine the relationship between diet and nutritional status at a given point in time, and cohort studies, which follow patients over the long term to understand the impact of interventions. Randomized controlled trials (RCTs), which are considered the most relevant to determine the direct effect of dietary changes, were also included. To ensure the quality of the evidence, only studies that used objective measures of nutritional status such as muscle mass, markers of malnutrition or biochemical tests and validated assessment tools were selected. A strength of this review is the analysis of the mechanisms by which diet can influence nutritional status, providing insight into the pathophysiological effects. Also, the inclusion of a large number of articles, including recent works, allows for a comprehensive update of existing knowledge. However, the diversity of methodologies and variable quality of studies pose a challenge, preventing a meta-analysis and limiting the generalizability of the results. Even so, the information obtained provides a solid foundation for future research in oncology nutrition and helps to better understand how dietary interventions can support patients.

## 4. Limitations

This review has several limitations that should be acknowledged. First, study selection and data extraction were performed by a single investigator, which may increase the risk of selection and extraction bias, despite the use of predefined eligibility criteria and a standardized data extraction template. Second, due to the heterogeneity of study designs, populations, nutritional assessment methods, and reported outcomes, a quantitative meta-analysis was not feasible, and the results were summarized descriptively. Third, a formal standardized risk of bias assessment tool was not applied; instead, methodological quality was assessed qualitatively, which may limit the ability to compare the strength of evidence across studies. These limitations should be considered when interpreting the results and drawing conclusions.

## 5. Conclusions

Given the limitations of conventional treatments for maintaining nutritional status in patients with digestive cancers, additional dietary interventions may represent a promising strategy for the prevention and management of cancer-associated malnutrition. Existing evidence suggests that strategies such as protein supplementation, adequate caloric intake, and the use of essential fatty acids may support the maintenance of muscle mass, reduce weight loss, shorten the length of hospital stay, and improve quality of life.

However, results are often inconsistent due to methodological heterogeneity, small sample sizes, and limited control of confounding factors. Interventions appear to be more effective when they are personalized and applied over sufficiently long periods to produce measurable effects on body mass and nutritional markers.

To provide the most comprehensive picture of the literature, we included both observational and cross-sectional studies, as well as randomized controlled trials and meta-analyses. Cross-sectional studies provide relevant information on the prevalence of malnutrition, dietary trends, and association with clinical parameters, although they do not allow for causal inferences. Consequently, conclusions regarding the effects of dietary interventions should be interpreted with caution.

This review has several important limitations. Most of the included studies were observational, which limits the establishment of causal relationships. Heterogeneity between cancer types, nutritional assessment methods, and duration of interventions reduced the comparability of results. Publication bias and lack of details on patient adherence or intervention composition may also influence the conclusions. Despite an extensive search, some relevant studies may have been missed. Well-designed randomized clinical trials are needed to confirm the precise effects of dietary interventions and to optimize nutritional strategies in patients with digestive cancers.

## Data Availability

All data related to this manuscript are available in the form of tables and figures in the manuscript.

## Supplementary Materials

The following supporting information can be downloaded at: https://www.mdpi.com/article/10.3390/biomedicines14010240/s1, Table S1: PRISMA checklist. Reference [131] are cited in the Supplementary Materials.
