# Supplementary material for: Effects of Dietary Interventions on Nutritional Status in Patients with Gastrointestinal Cancers: A Systematic Review
Source: Biomedicines. 2026 Jan 21;14(1):240. doi: 10.3390/biomedicines14010240 (PMC12839109; doi:10.3390/biomedicines14010240)
Supplement: Supplementary file 1 [file biomedicines-14-00240-s001.zip › biomedicines-4072088-PRISMA_2020_checklist.pdf]

PRISMA 2020 Checklist

Section and Topic	Item #	Checklist item	Location where item is reported
**TITLE**			
Title	1	Identify the report as a systematic review.	Chapter 2 Methods
**ABSTRACT**			
Abstract	2	See the PRISMA 2020 for Abstracts checklist.	Provided at the next material upload
**INTRODUCTION**			
Rationale	3	Describe the rationale for the review in the context of existing knowledge.	Cancer reflects a complex pathology characterized by a chaotic and uncontrolled proliferation of cells in a tissue or organ, leading to the appearance of a cancerous neoplasm. Digestive cancers are among the most common types of cancer. Malnutrition is a multifactorial condition, which includes a combination of varying degrees of undernutrition, overnutrition and the presence of swelling, leading to the alteration of physical and mental functions with an unfavorable impact on the evolution of the disease and the response to treatment
Objectives	4	Provide an explicit statement of the objective(s) or question(s) the review addresses.	The objective of this review was to evaluate the impact of dietary interventions on the nutritional status in patients with digestive neoplasms prone to malnutrition, based on cross-sectional and cohort studies
**METHODS**			
Eligibility criteria	5	Specify the inclusion and exclusion criteria for the review and how studies were grouped for the syntheses.	*2.1. Study eligibility: inclusion and exclusion criteria*<br>The selection of articles was carried out based on pre-established criteria, presented both descriptively and in tabular format.<br>Inclusion Criteria:<br>1. Articles published in English;<br>2. Studies conducted on adult population (≥18 years);<br>3. Observational studies (cross-sectional, cohort) and interventional studies (RCT);<br>4. Research that evaluated the relationship between malnutrition and survival;<br>5. Studies that investigated sarcopenia and cachexia;<br>6. Studies that analyzed the role of nutrition in the management of adverse effects associated with oncological treatments;<br>7. Studies that used validated instruments to measure nutritional status;<br>8. Articles that examined enteral and parenteral nutrition.<br>Exclusion criteria:<br>1. Publications in languages other than English;<br>2. Studies for which the full text was not available;<br>3. Research that included participants under 18 years of age;<br>4. Experimental animal studies;

Section and Topic	Item #	Checklist item	Location where item is reported
			5. In vitro studies.
Information sources	6	Specify all databases, registers, websites, organisations, reference lists and other sources searched or consulted to identify studies. Specify the date when each source was last searched or consulted.	the PubMed, MDPI, ResearchGate and ScienceDirect databases. August 2025
Search strategy	7	Present the full search strategies for all databases, registers and websites, including any filters and limits used.	2.2. Search strategy and article selection<br>[...]<br>The selection strategy was limited to research articles published in English between 2009 and 2025. The systematic search initially identified 14,503 articles. After removing 5,703 duplicates, 8,800 articles were subjected to title and abstract screening. Of these, 8,685 were excluded, leaving 115 articles for full-text evaluation. Following the application of the eligibility criteria, 35 articles were excluded for various reasons (inappropriate population, inappropriate study type, lack of reporting of nutritional interventions or lack of full access). Finally, 80 studies were included in the systematic review. The selection process followed the steps specified in the PRISMA 2020 flowchart presented in (Figure 2).
Selection process	8	Specify the methods used to decide whether a study met the inclusion criteria of the review, including how many reviewers screened each record and each report retrieved, whether they worked independently, and if applicable, details of automation tools used in the process.	The selection process followed the steps specified in the PRISMA 2020 flowchart presented in (Figure 2).
Data collection process	9	Specify the methods used to collect data from reports, including how many reviewers collected data from each report, whether they worked independently, any processes for obtaining or confirming data from study investigators, and if applicable, details of automation tools used in the process.	*2.3. Data extraction*<br>All necessary information was obtained from the articles included in the systematic review. The extracted data covered the following aspects: study design (cross-sectional, cohort, observational, randomized clinical trials), population characteristics (age, sex, number of participants, nationality/country, diagnosis), monitored parameters (BMI, anthropometric measurements), type of nutrition administered (including enteral nutrition) and outcomes.<br><br>The data extraction process was performed independently by a single researcher.
Data items	10a	List and define all outcomes for which data were sought. Specify whether all results that were compatible with each outcome domain in each study were sought (e.g. for all measures, time points, analyses), and if not, the methods	2.2. Search strategy and article selection

Section and Topic	Item #	Checklist item	Location where item is reported
		used to decide which results to collect.	
	10b	List and define all other variables for which data were sought (e.g. participant and intervention characteristics, funding sources). Describe any assumptions made about any missing or unclear information.	Not applicable
Study risk of bias assessment	11	Specify the methods used to assess risk of bias in the included studies, including details of the tool(s) used, how many reviewers assessed each study and whether they worked independently, and if applicable, details of automation tools used in the process.	Not applicable
Effect measures	12	Specify for each outcome the effect measure(s) (e.g. risk ratio, mean difference) used in the synthesis or presentation of results.	Not applicable
Synthesis methods	13a	Describe the processes used to decide which studies were eligible for each synthesis (e.g. tabulating the study intervention characteristics and comparing against the planned groups for each synthesis (item #5)).	*2.1. Study eligibility: inclusion and exclusion criteria*<br>The selection of articles was carried out based on pre-established criteria, presented both descriptively and in tabular format.
	13b	Describe any methods required to prepare the data for presentation or synthesis, such as handling of missing summary statistics, or data conversions.	Not applicable
	13c	Describe any methods used to tabulate or visually display results of individual studies and syntheses.	The selection process followed the steps specified in the PRISMA 2020 flowchart presented in (Figure 2).
	13d	Describe any methods used to synthesize results and provide a rationale for the choice(s). If meta-analysis was performed, describe the model(s), method(s) to identify the presence and extent of statistical heterogeneity, and software package(s) used.	Not applicable
	13e	Describe any methods used to explore possible causes of heterogeneity among study results (e.g. subgroup analysis, meta-regression).	Not applicable
	13f	Describe any sensitivity analyses conducted to assess robustness of the synthesized results.	Not applicable
Reporting bias	14	Describe any methods used to assess risk of bias due to missing results in a synthesis	Not applicable

Section and Topic	Item #	Checklist item	Location where item is reported
assessment		(arising from reporting biases).	
Certainty assessment	15	Describe any methods used to assess certainty (or confidence) in the body of evidence for an outcome.	3. Discussion<br>[....]<br>To ensure the quality of the evidence, only studies that used objective measures of nutritional status such as muscle mass, markers of malnutrition or biochemical tests and validated assessment tools were selected. A strength of this review is the analysis of the mechanisms by which diet can influence nutritional status, providing insight into the pathophysiological effects. Also, the inclusion of a large number of articles, including recent works, allows for a comprehensive update of existing knowledge
**RESULTS**			
Study selection	16a	Describe the results of the search and selection process, from the number of records identified in the search to the number of studies included in the review, ideally using a flow diagram.	Page 4 of manuscript
	16b	Cite studies that might appear to meet the inclusion criteria, but which were excluded, and explain why they were excluded.	Not applicable
Study characteristics	17	Cite each included study and present its characteristics.	Not applicable
Risk of bias in studies	18	Present assessments of risk of bias for each included study.	Not applicable
Results of individual studies	19	For all outcomes, present, for each study: (a) summary statistics for each group (where appropriate) and (b) an effect estimate and its precision (e.g. confidence/credible interval), ideally using structured tables or plots.	Not applicable
Results of syntheses	20a	For each synthesis, briefly summarise the characteristics and risk of bias among contributing studies.	Not applicable
	20b	Present results of all statistical syntheses conducted. If meta-analysis was done, present for each the summary estimate and its precision (e.g. confidence/credible interval) and measures of statistical heterogeneity. If comparing groups, describe the direction of the effect.	Not applicable
	20c	Present results of all investigations of possible causes of heterogeneity among study results.	Discussion: [...] This subsection reviewed studies that assessed how dietary interventions

PRISMA 2020 Checklist

Section and Topic	Item #	Checklist item	Location where item is reported
			influence nutritional status in patients with digestive cancers. The results are not uniform: some studies show benefits, while others do not show significant effects. The review includes both cross-sectional studies, which examine the relationship between diet and nutritional status at a given point in time, and cohort studies, which follow patients over the long term to understand the impact of interventions. Randomized controlled trials (RCTs), which are considered the most relevant to determine the direct effect of dietary changes, were also included.
	20d	Present results of all sensitivity analyses conducted to assess the robustness of the synthesized results.	Not applicable
Reporting biases	21	Present assessments of risk of bias due to missing results (arising from reporting biases) for each synthesis assessed.	Not applicable
Certainty of evidence	22	Present assessments of certainty (or confidence) in the body of evidence for each outcome assessed.	Discussion [...] To ensure the quality of the evidence, only studies that used objective measures of nutritional status such as muscle mass, markers of malnutrition or biochemical tests and validated assessment tools were selected. A strength of this review is the analysis of the mechanisms by which diet can influence nutritional status, providing insight into the pathophysiological effects.
**DISCUSSION**			
Discussion	23a	Provide a general interpretation of the results in the context of other evidence.	Provided
	23b	Discuss any limitations of the evidence included in the review.	Provided
	23c	Discuss any limitations of the review processes used.	Provided
	23d	Discuss implications of the results for practice, policy, and future research.	Provided
**OTHER INFORMATION**			
Registration and protocol	24a	Provide registration information for the review, including register name and registration number, or state that the review was not registered.	Not applicable
	24b	Indicate where the review protocol can be accessed, or state that a protocol was not prepared.	Not applicable
	24c	Describe and explain any amendments to information provided at registration or in the protocol.	Not applicable

PRISMA 2020 Checklist

Section and Topic	Item #	Checklist item	Location where item is reported
Support	25	Describe sources of financial or non-financial support for the review, and the role of the funders or sponsors in the review.	Support was provided by the University of Oradea through the grant competition "Scientific Research of Excellence Related to Priority Areas with Capitalization through Technology Transfer: INO TRANSFER—UO". Project no. 258/2022.
Competing interests	26	Declare any competing interests of review authors.	The authors declare no conflict of interest.
Availability of data, code and other materials	27	Report which of the following are publicly available and where they can be found: template data collection forms; data extracted from included studies; data used for all analyses; analytic code; any other materials used in the review.	Data will be available upon request.

From: Page MJ, McKenzie JE, Bossuyt PM, Boutron I, Hoffmann TC, Mulrow CD, et al. The PRISMA 2020 statement: an updated guideline for reporting systematic reviews. BMJ 2021;372:n71. doi: 10.1136/bmj.n71. This work is licensed under CC BY 4.0. To view a copy of this license, visit https://creativecommons.org/licenses/by/4.0/
